# Agreement between original and Rasch-approved neck disability index

**DOI:** 10.1186/s12874-020-01069-w

**Published:** 2020-07-03

**Authors:** Ze Lu, Joy C. MacDermid, Goris Nazari

**Affiliations:** 1 Clinical Research Lab, Hand and Upper Limb Center, St Joseph’s Health Care, London, ON Canada; 2 The School of Rehabilitation Science, McMaster University, Hamilton, ON Canada; 3 School of Physical Therapy, Health and Rehabilitation Science, Western University, London, ON Canada; 4 The Collaborative Specialization Musculoskeletal Health Research (CMHR), The Bone and Joint Institute, Western University, London, ON Canada

**Keywords:** Neck pain, Outcome measure, Agreement analysis, Bland-Altman, Rasch analysis

## Abstract

**Background:**

Given the high prevalence of neck pain, the neck disability index (NDI) has been used to evaluate patient status and treatment outcomes. Modified versions were proposed as solutions to measurement deficits in the NDI. However, the original 10-item NDI was scored out of 50 and is still the most frequently administered version. Examining the extent of agreement between traditional and Rasch-based versions using Bland-Altman (B&A) plots will inform our understanding of score differences that might rise from using different versions. Therefore, the objective of current study was to describe the extent of agreement between different versions of NDI.

**Methods:**

The current study was a secondary data analysis. The study data was compiled from two prospectively collected data sources. We performed a comprehensive literature search to identify Rasch approved NDI within four databases including Embase, Medline, PubMed, and Google Scholar. Alternate forms and scorings were compared to each other and to the standard NDI. We graphed B&A plots and calculated the mean difference and the 95% limits of agreement (LoA; ±1.96 times the standard deviation).

**Results:**

Two Rasch approved alternative versions (8 vs 5 items) were identified from 303 screened publications. We analyzed data from 201 (43 males and 158 females) patients attending community clinics for neck pain. We found that the mean difference was approximately 10% of the total score between the 10-item and 5-item (− 4.6 points), whereas the 10-item versus 8-item and 8-item versus 5-item had smaller mean differences (− 2.3 points). The B&A plots displayed wider 95% LoA for the agreement between 10-item and 8-item (LoA: − 12.0, 7.4) and 5-item (LoA: − 14.9, 5.8) compared with the LoA for the 8-item and 5-item (LoA: − 7.8, 3.3).

**Conclusion:**

Two Rasch-based NDI solutions (8 vs 5 items) which differ in number of items and conceptual construction are available to provide interval level scoring. They both provide scores that are substantially different from the ordinal NDI, which does not provide interval level scoring. Smaller differences between the two Rasch solutions exist and may relate to the items included. Due to the size and unpredictable nature of the bias between measures, they should not be used interchangeably.

## Background

Neck pain is considered a notable social burden and has a high point prevalence (33%) within the adult population, and nearly 70% of people will experience neck pain at some point during their lifetime [4, 7, 8, 12, 16]. Clinical decision-making requires monitoring the treatment effect (improvement or deterioration) from both clinician and patient perspectives. The first patient-reported outcome measure (PROM) that assessed pain and disability in participants with neck pain was published in 1991 – the 10-item version of neck disability index (NDI-10 )[22]. The NDI-10 is the most studied neck-related PROM as it has been cited and applied in more than 300 publications [21]. It has been used widely in surgical treatment, injection therapies, physical therapy, as well as within exercise and research context [15, 16, 21]. Both a systematic review [16] and an overview [3] have reviewed a large volume of psychometric evidence on NDI with most studies suggesting that the NDI-10 has excellent classical psychometric properties, while a few studies have raised concerns about its factor structure, item relevance or scaling. The original version of the NDI-10 has been translated into 22 languages versions [9, 21].

The NDI-10 was developed as a unidimensional instrument assessing neck disability, with this as a fundamental requirement for using a single summary score [18–20]. The NDI-10 contains 10 items including pain intensity, personal care, lifting, reading, headaches, concentration, work, driving, sleeping, and recreation. Each item has 6 response options ranging from 0 to 5, where 0 represents the best situation and 5 represents the worst. Individual scores are summed to derive a total score from 0 to 50 with higher scores indicating more serious level of disability. Multiple items ask about pain and function together, which we consider to be more representative of the construct of pain-related functional interference. Through the ﻿problem elicitation technique (PET), others have concluded that the NDI-10 is a multidimensional scale that measures symptoms, impairments, and disabilities (work, recreation) [13].

Previous researchers have examined the NDI-10 using factor analysis, qualitative interview, and construct analysis under the classical test theory (CTT) [14]. Gabel et al. [10] concluded that the NDI-10 is a one-factor model confirmed by confirmatory factor analysis in a homogenous population with neck pain. However, others identified 2 factors using a principal component analysis [25].

Rasch analysis based on item response theory (IRT) and Rasch modelling enables examination of unidimensionality and interval level of scaling, and can lead to a transformation strategy to convert an ordinal score to interval scaling, which can validate the use of a total sum score [5]. Where outcome measures are not developed using Rasch modelling, they can retrospectively be evaluated for fit to the Rasch model which often result in suggested modifications needed to obtain fit. Several studies have inspected the NDI-10 using Rasch analysis and found violations of Rasch basic assumptions [10, 20, 24]. They offered solutions which included exclusion of misfit items and new coding algorithms. Although modified versions of NDI have been constructed that are conceptually and statistically sound, uptake has been limited and the traditional NDI-10 is still commonly used. Studies to date have focused on defining modified versions with better measurement properties but have not defined the extent to which these new versions differ from the traditional NDI-10 scoring outside of the development data set. Examining the amount of agreement between traditional and Rasch-based versions of the NDI using Bland-Altman (B&A) plots will inform our understanding of how these scores might differ [1, 2, 17].

Therefore, the objective of current study was to describe the extent of agreement between different versions of NDI in a sample of patients attending community clinics for neck pain.

## Methods

### Study design

The current study was a secondary data analysis where the study data was compiled from two prospectively collected data source. Both studies received ethical approval (McMaster Research Ethics Board (MREB) #03–145 and Hamilton Integrated Research Ethics Board (HiREB) #13–300) and all participants provided written, signed consent. Participants were recruited from community clinics presenting with neck pain in Hamilton, ON Canada through paper and online based survey.

### Information source

We performed a comprehensive literature search to identify Rasch analyses of the NDI within four databases including Embase, Medline, PubMed, and Google Scholar. Search keywords were set as neck disability index, NDI, Rasch analysis, structural validity, construct validity. The search year range was limited until January 2020. Details of search strategies were presented in Appendix 1.

### Study selection

An independent reviewer (ZL) performed the systematic electronic searches in all the databases. ZL also identified and removed the duplicate studies. The independent reviewer then carried out the screening of the titles/abstracts and identifying the full text articles. One author [JMacD] randomly reviewed 50% of the articles and discussed the disagreement with the first author to determine the final article eligibility.

### Acceptable Rasch solutions

We included studies that applied the Rasch model to evaluate the structural validity of NDI. The score transformation algorithm was obtained if the revised version achieved an acceptable level of model fit identified by the eligibility criteria. According to assumptions of the Rasch theory, we defined the acceptable fit of the Rasch model as follows:

1. Unidimensionality was confirmed.

E.g. In studies using the Rasch analysis software, RUMM2030 (Rumm Laboratory, Australia) we used the common criterion that acceptable unidimensionality was present if the number of significant tests was less than 5% of the overall paired sample t-tests [19].

2. Overall test-fit statistic was examined by the Chi-square test; a non-significant *p*-value was acceptable.
3. Where response categories had disordered thresholds, strategies such as collapsing the adjacent response options were used as corrective actions, and the rescoring structure was reported and used to calculate revised NDI scores.
4. There was no differential item functioning (DIF), either uniform or non-uniform DIF, in the revised version.
5. Local dependency was assessed, and scale amendments taken where appropriate.
6. An appropriate level of the person separation index was demonstrated e.g. (PSI > 0.7)

### Statistical procedures

The scores of alternate versions were computed. The demographic statistics of the sample including age, sex, total score of all included versions of NDI were described by mean, standard deviation (SD), median, interquartile range, minimum and maximum value. We performed the Wilcoxon signed rank test to perform a non-parametric comparison between NDI scores since the total score of NDI-10 was computed from ordinal scale.

### Agreement of Rasch solutions

The normal distribution of mean differences of all three comparisons were inspected by the histogram. Using the B&A plots, we summarized the individual agreement between each of the identified NDI versions by the mean difference and the 95% limits of agreement (LoA; ±1.96 times the standard deviation).

To test the average agreement and differences between each NDI score, we examined the mean differences by one-sample t-test [11]. We reported the sample size for each comparison, the degree of freedom, mean differences with *p*-value and 95% confidence interval (CI), standard error of differences (SE).

Transformations including logarithmic and linear transformations were applied to normalize the non-uniform pattern of the bias on the plot. For instance, when the B&A plot shows a linear relationship between differences and means, (the differences measurement bias start with negative value and then becomes positive while the magnitude of the mean increases), we can regress differences between the methods (D) on the average of the two methods (A) by D = b1 × A + b0. The 95% LoA for the regression should build on the SD of the residual (SDres) from the established model (±1.96 times SDres) [1].

All analysis was performed by IBM SPSS statistics, Version 25.0 (IBM Corporation, Armonk, NY). We considered a significance level of *p* ≤ 0.05 as statistically significant.

## Result

### Study selection and NDI version identification

**Fig. 1:**
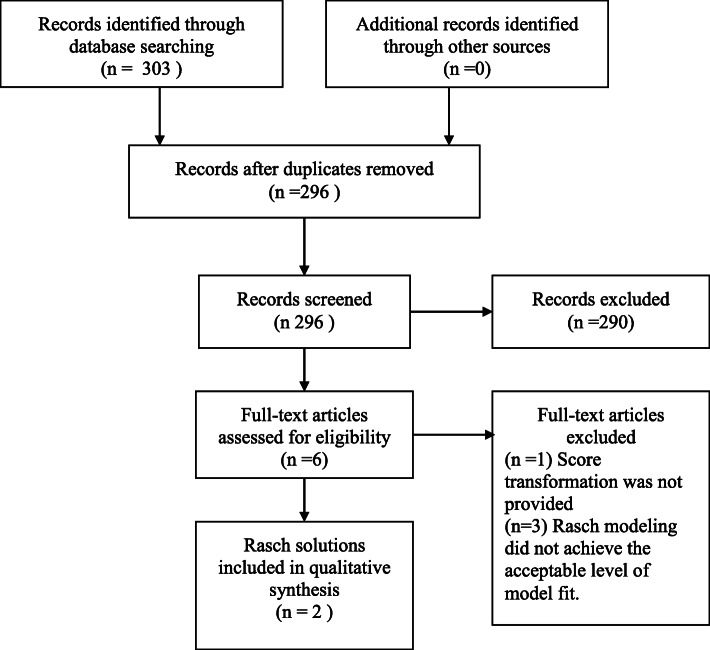
Flow Diagram of study selection results based on PRISMA guideline

Initially, our search yielded 303 publications. After removing the duplications, 296 articles were left. Six studies were then selected for full text review after title and abstract review. Of these, two Rasch solutions that met the study criteria were identified from 2 individual studies including a 8-item version NDI (NDI-8) developed by Van Der Velde and colleagues [20] which was based on Rasch criteria, and a 5-item version NDI (NDI-5) developed by Walton and MacDermid [24] based on conceptual and Rasch criteria [24]. This allowed 3 B&A comparisons (NDI-10 vs. NDI-8, NDI-10 vs.NDI-5, and NDI-8 vs. NDI-5). The flowchart of studies through the selection process is displayed in Fig. 1.

### Ordinal score transformation

Three NDI scores were calculated for each participant. The first NDI score was derived from the original ordinal scale (maximum of 50 )[21]. We calculated second set of NDI scores according to the 8 item Rasch solution provided by Van Der Velde and collogues [20], where 2 items (headache and lifting) were removed and then, the ordinal scores were transferred to linear score with the maximum value of 50. For third score transformation, two steps were taken to derive the total score as recommended in a study that considered both conceptual issues and Rasch findings [24]. Firstly, 5 functional items regarding person care, concentration, working, driving, and recreation were kept into the total score calculation. A rescoring strategy, was then used to remedy the disordered threshold of driving related item [24]. The original score of responses (012345) was re-coded by collapsing the fourth and fifth options (012334), while the original structure (012345) was retained for other 4 items. Therefore, the maximum total score of NDI 5-item version was 24 on the ordinal scale. This score was transformed in to an equivalent ranging from 0 to 50 to enable the direct comparisons [24]. Please see Appendix 2 for a summary of transformations.

### Sample

**Fig. 2:**
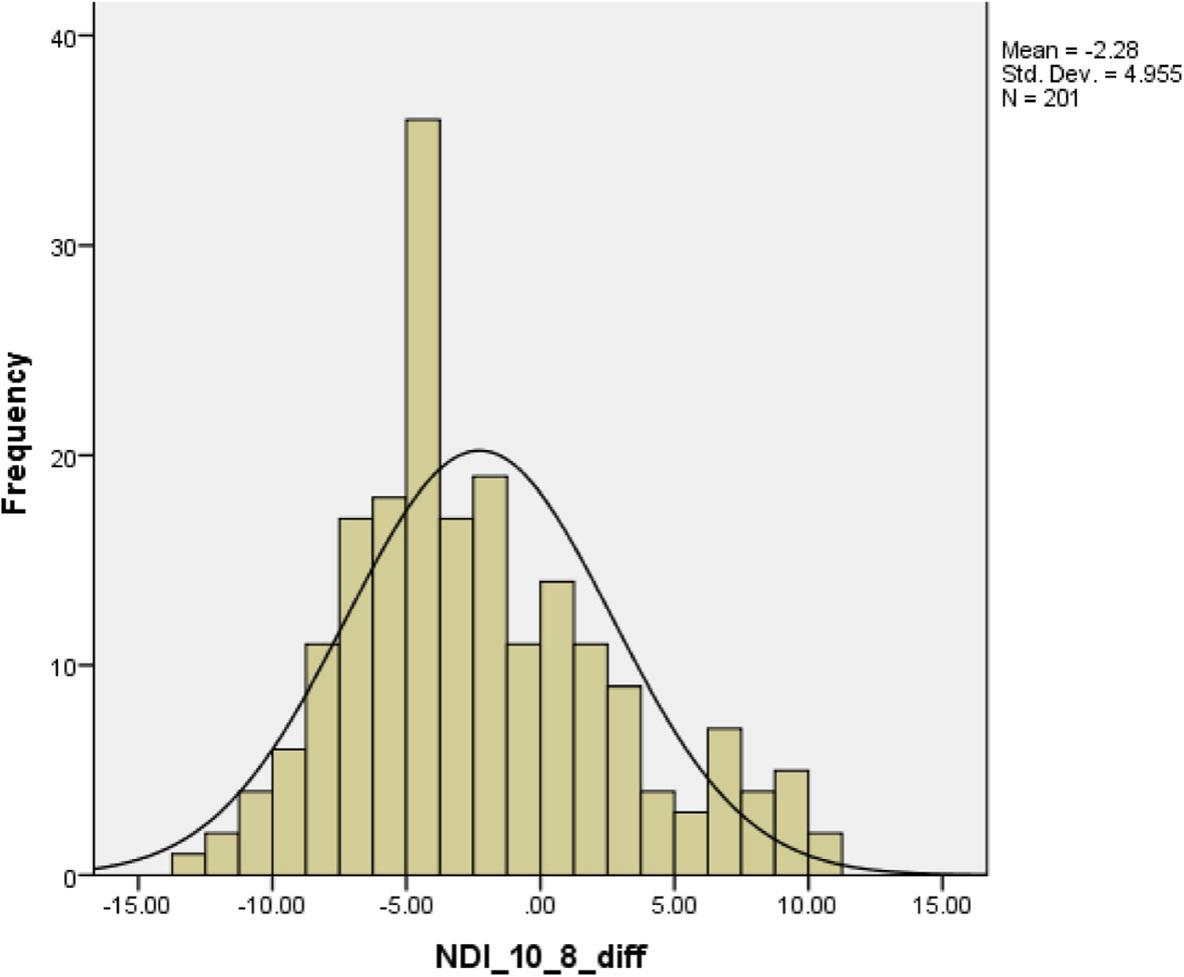
Histogram of the difference comparing NDI 10-item total score with NDI 8-item total score. NDI: neck disability index

**Fig. 3:**
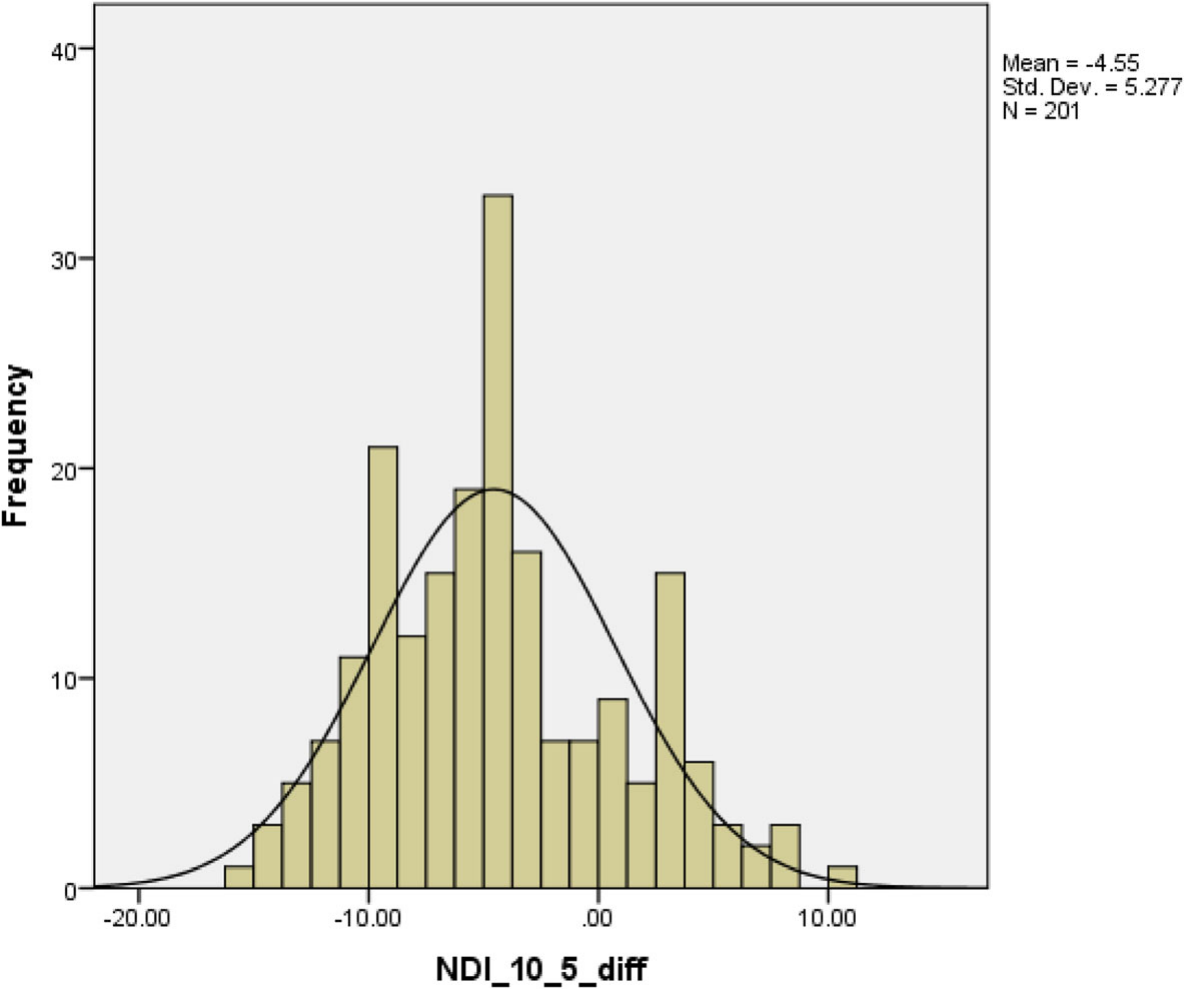
Histogram of the difference comparing NDI 10-item total score with NDI 5-item total score. NDI: neck disability index

**Fig. 4:**
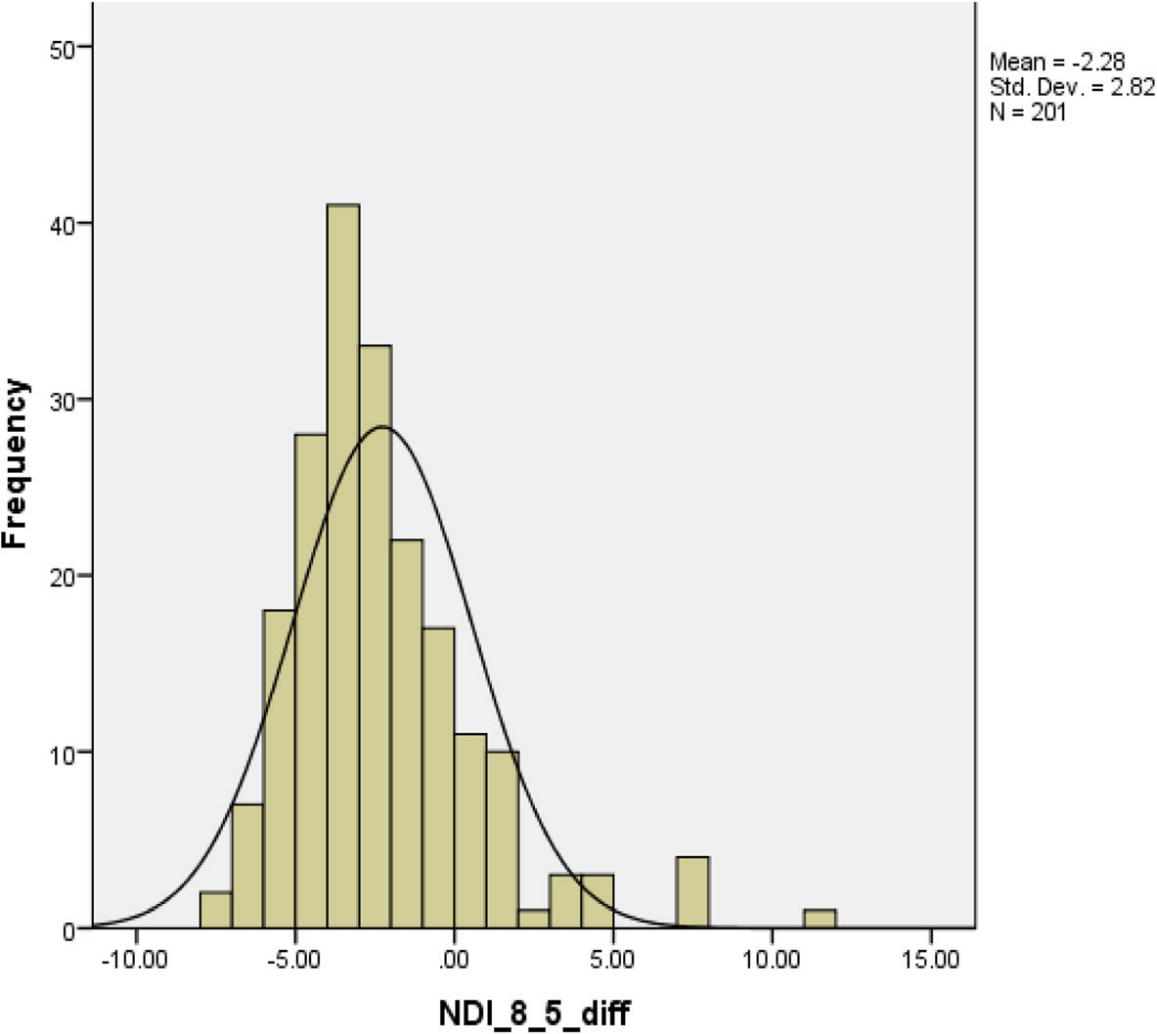
Histogram of the difference comparing NDI 8-item total score with NDI 5-item total score. NDI: neck disability index

Table 1 describes the demographic information including age, pain intensity, total scores of NDI-10, NDI-8, and NDI-5 and stratified by sex. Thirty-one subjects experienced injury or trauma related neck-pain including car accident, sports injury, and fall. Other conditions leading to neck pain were arthritis, pinched nerves, and disc problems. The normal distribution of the mean differences of comparisons were confirmed by inspecting the histogram. See Figs. 2, 3, and 4. The Wilcoxon signed rank test revealed statistically significant differences between total scores from each two NDI versions (NDI-10 vs. NDI-8, NDI-10 vs. NDI-5, and NDI-8 vs. NDI-5). See Table 2.

**Table 1:** Demographic characteristic of the sample

	Male (*N* = 43)		Female (*N* = 158)		Total (*N* = 201)		Median (IQR)
	Mean (SD)	Range, min-max	Mean (SD)	Range, min-max	Mean (SD)	Range, min-max	
**Age, year**	49.2 (12.2)	19–74	45.7 (12.8)	19–74	46.5 (12.8)	19–74	NA
**Pain intensity**	2.0 (1.4)	0–5	2.1 (1.2)	0–5	2.1 (1.3)	0–5	NA
**NDI-10**	14.6 (10.7)	2–44	17.0 (9.8)	0–41	16.4 (10.0)	0–44	15.0 (14.0)
**NDI-8**	17.8 (7.0)	0–33	19.0 (6.0)	0–31.5	18.4 (6.2)	0–32.3	19.2 (8.2)
**NDI-5**	20.1 (8.3)	0–35	21.2 (7.6)	0–33	21.0 (7.8)	0–35.0	22.0 (11.0)

NDI-10: The total score of NDI 10-item (original) version on ordinal scale with maximum of 50 points

NDI-8: The total score of NDI 8-item version on linear scale with maximum of 50 points

NDI-5: The total score of NDI 5-item version on linear scale with maximum of 50 points

*SD* standard deviation, *Min* minimum, *Ma* maximum, *IQR* Interquartile range

**Table 2:** Bland-Altman statistics and non-parametric comparisons by Wilcoxon signed rank test

Comparison	Sample size	Degree of freedom	Individual agreement				Average agreement	Wilcoxon signed rank test
			Mean of difference (d) with (95% CI)	SD of difference	Upper LoA d + 1.96SD	Lower LoA d-1.96SD	SE	
NDI-10 vs. NDI-8	201	200	−2.3* (−3.0 - -1.6)	5.0	7.4	−12.0	0.4	*P* < 0.001*
NDI-10 vs. NDI-5	201	200	−4.6* (−5.3 - -3.8)	5.3	5.8	−14.9	0.5	*P* < 0.001*
NDI-8 vs. NDI-5	201	200	−2.3* (−2.7 - -1.9)	2.8	3.3	−7.8	0.2	*P* < 0.001*

* significant as *p* < 0.001

NDI-10: The total score of NDI 10-item (original) version on ordinal scale with maximum of 50 points

NDI-8: The total score of NDI 8-item version on linear scale with maximum of 50 points

NDI-5: The total score of NDI 5-item version on linear scale with maximum of 50 points

*SD* standard deviation

*SE* standard error base on the mean of difference

### Agreement of Rasch solutions

Table 2 demonstrated both average and individual agreement results of all three comparisons.

Through pairwise comparisons, we identified that the mean difference was approximately 10% of the total score between the NDI-10 and NDI-5 (− 4.6 points), whereas the NDI-10 versus NDI-8 and NDI-8 versus NDI-5had similar mean differences that were about half (− 2.3 points). We considered the NDI-10 as the reference method during comparisons, negative mean differences indicating that both NDI-8 and NDI-5 systematically scored higher than standard NDI-10 The B&A plots displayed wider 95% LoA for the agreement between NDI-10 and NDI-8 (− 12.0, 7.4) and NDI-5 (− 14.9, 5.8) compared with the agreement between the NDI-8 and NDI-5 (− 7.8, 3.3).

**Fig. 5:**
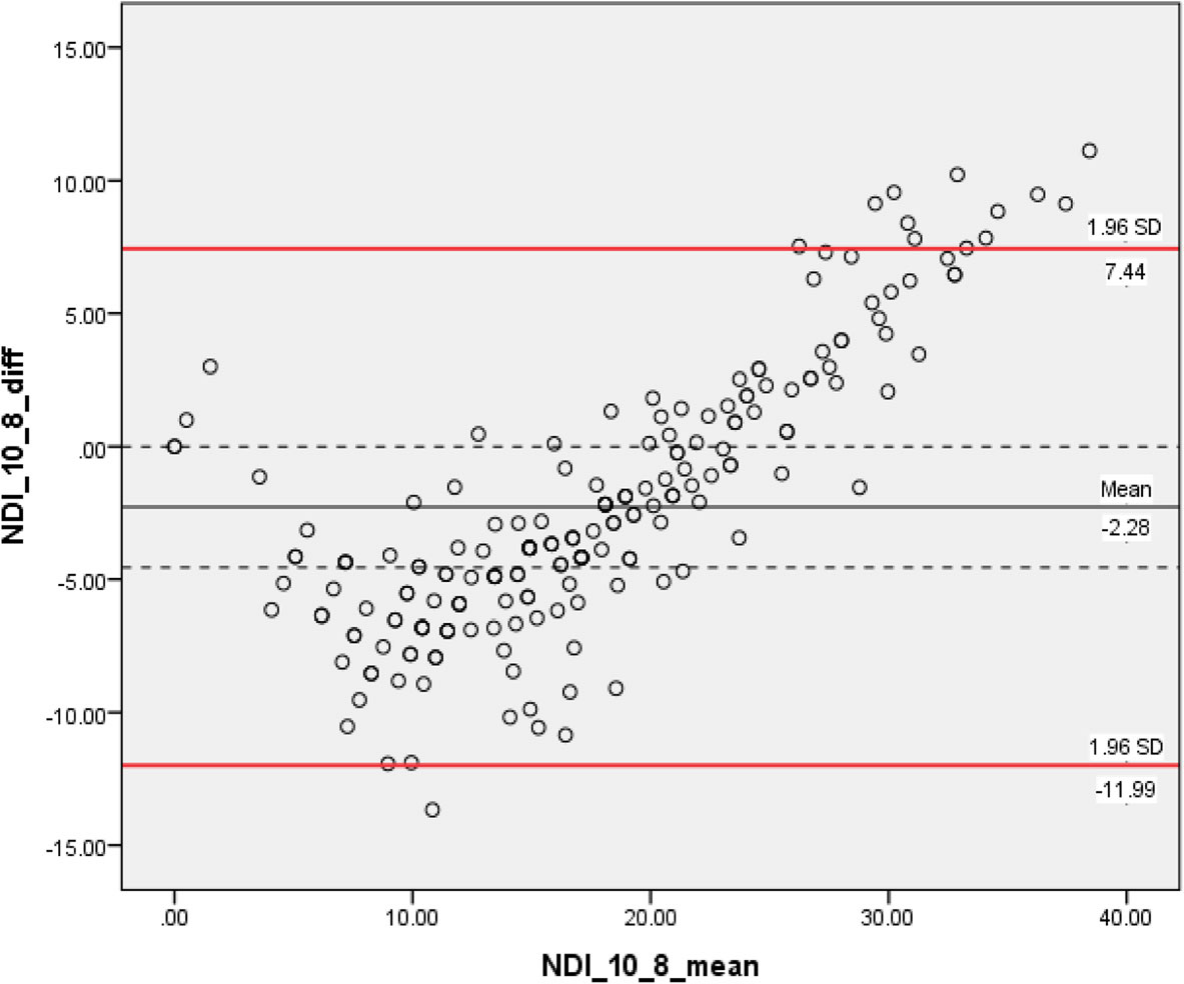
Bland–Altman plots displaying 95% LoA in pair-wise comparison between NDI 10-item with NDI 8-item version. LoA: limits of agreement. NDI: neck disability index

**Fig. 6:**
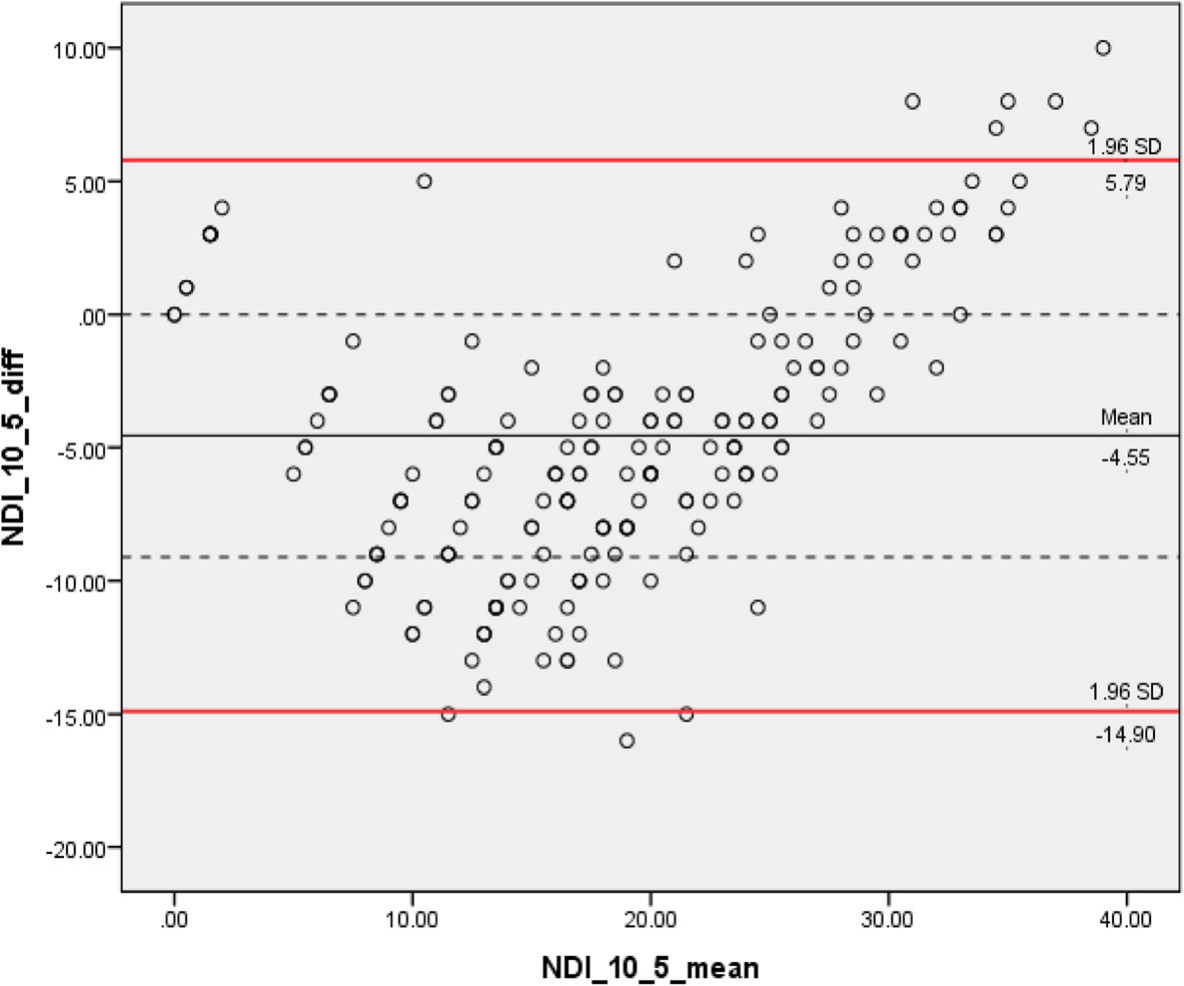
Bland–Altman plots displaying 95% LoA in pair-wise comparison between NDI 10-item with NDI 5-item version. LoA: limits of agreement. NDI: neck disability index

**Fig. 7:**
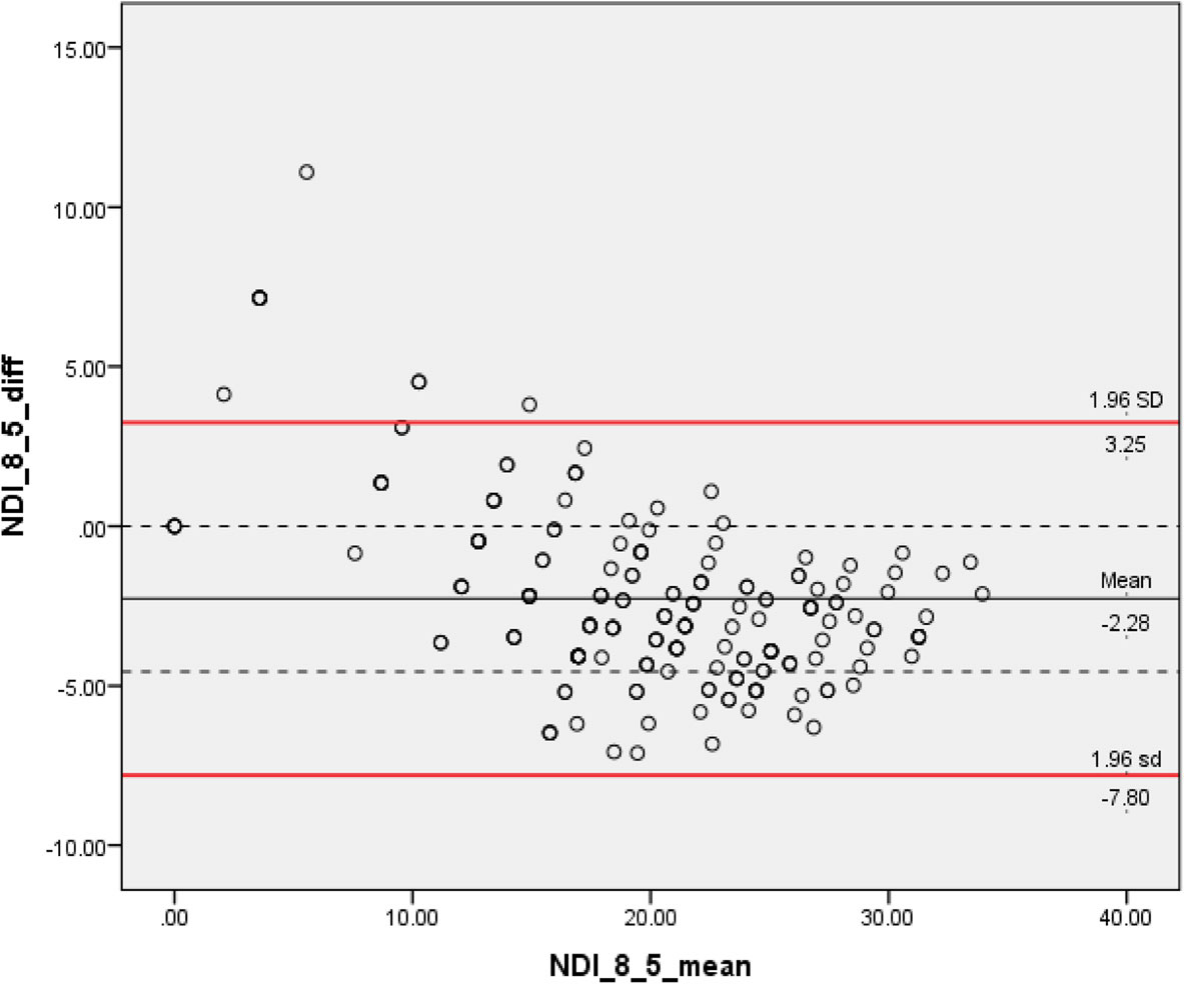
Bland–Altman plots displaying 95% LoA in pair-wise comparison between NDI 8-item with NDI 5-item version. LoA: limits of agreement. NDI: neck disability index

Through visual inspection of the Bland-Altman plot, the bias between NDI-10 and NDI-8 tended to be in opposite directions at different point in the scale range, as negative value of differences predominated in the lower end (before scores of 20) and positive values predominated in the high end of the scale (between 20 and 40). A similar trend was identified in the comparison between NDI-10 and NDI-5. However, such patterns were not present in the plot comparing NDI-8 with NDI-5. Please see Figs. 5, 6, 7.

**Fig. 8:**
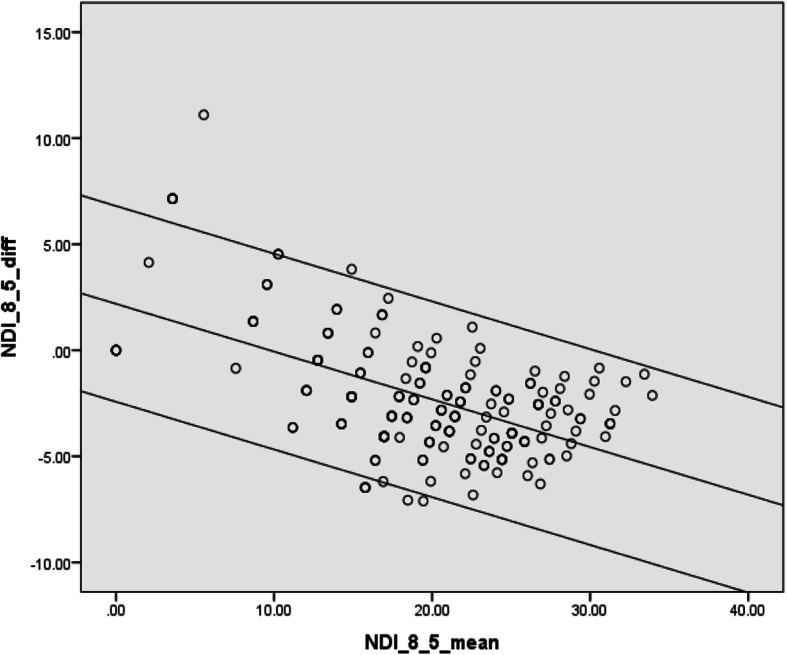
Bland–Altman plots displaying 95% LoA in regression between NDI 8-item with NDI 5-item version as this varies across the range of the scores. LoA: limits of agreement. NDI: neck disability index

The linear relationship on the B&A plot comparing NDI-8 with NDI-5was confirmed by the simple linear regression eq. D = − 0.2 × A + 2.2 with a significant *p* value for the over model and regression coefficient (*p* < 0.001) [1]. We then plotted 95% LoA based on the SDres which was equal to 2.4 from the regression model. The new upper and lower limited was constructed as D = − 0.2 × A + 2.189 ± 1.96 × 2.4. See Fig. 8.

## Discussion

We identified two Rasch approved versions of the NDI (NDI-8 and NDI-5) through a comprehensive literature review and revealed disagreements in score results within versions (NDI-10 vs. NDI-8 and NDI-5) using B&A plot analysis [11, 20, 24].. Such significant differences within versions were identified in non-parametric group comparisons. The wide range of the 95% LoA established surrounding the point estimate of the agreement would threaten the interchangeable application of different versions. When compared the traditional NDI-10 with the 8 items Rasch approved version, a difference of ranging from − 12.0 to 7.4 units accounting for nearly 15 to 25% of the total score was important for a measurement of 50 units, since 9 units of change would significantly influence the classification of the disability level [21]. For example, a participant who obtained a score of 20 on the traditional NDI-10 would be considered to have moderate level of neck disability. However, the LoAs between Rasch versions suggest that scores might fall within the mild or severe level a range from − 12.0 to 7.4 units. This reflects the extent of misclassification error that might occur on the basis of scoring. The bias between versions was even larger 30% (− 14.9 for lower limit) when comparing the NDI-10 with the NDI-5. The differences between NDI-8 and NDI-5 were uniform after linear transformation and were smaller than the discordance between the traditional and Rasch scored versions, with a mean variation of 4.7 units (10% of the total score). This smaller difference likely reflects some benefits of a Rasch approach, but also some differences related to the number of items included. This smaller error still suggests that these measures cannot be used interchangeably. An advantage of the NDI-8 is that it 8 items may exhibit more range or stability than a 5-item version. Conversely, the NDI-5 is more focused conceptually since it focuses on function, and it reduces respondent burden. Head-to-head comparison of how these two versions performed in measuring clinical outcomes over time are needed to evaluate their relative utility.

The unstable variance in error patterns on B&A plot were problematic for comparing across Rasch versions, even though they had small error limits (− 2.3 and − 4.6). Through visual inspection, the direction of bias reverted when the scores approaching 20 points, approximately mid-range. Attempts including both logarithmic and linear transformation failed to normalize the bias pattern. The more extreme bias displayed at the upper and lower ends of the scale is reflective of the ordinal nature of the original 0–50 score, whereas the NDI-5 and NDI-8 have been linearly converted through the Rasch analytic process. This may explain why similar patterns were observed between the NDI-10 vs. NDI-8, and NDI-10 vs. NDI 5, but a different pattern was shown between the NDI-8 vs. NDI-5. Our data further illustrated that the original ordinal scale ranging from 0 to 50 should not be used in parametric statistical analyses, due to the violation of interval level scaling.

The differences between the NDI-8 and NDI-5 could be due to the variations in the retained items, both in terms of their content and the associated ‘difficulty’ level of the items. Firstly, fewer items are likely to result in a narrower measurement range coverage, and therefore the scale may be ‘stretched out’ when converted back to a 0–50 score. The smaller differences between the NDI-8 and NDI-5 may have been driven by methodologic differences in how these analyses were performed. In the NDI-8, the items (headache and lifting) were deleted based on Rasch findings drive by the goal of achieving optimal model fit [20]. For the 5-item version, the authors conducted a 2-stage process first deleting items for conceptual reasons and then proceeding to a Rasch analysis. The conceptual framework of the International Classification of Functioning, Disability and Health (ICF) was used to refine the item pool as to those that fit within the disability construct the symptom-based item such as pain intensity was removed at this stage [24]. This retention of symptoms in the NDI-8 and its exclusion from NDI-5 might explain the small systematic errors between the two Rasch-based versions. Researchers might select between these two versions based on these conceptual issues. For example, NDI-8 provides the evaluation of neck disability regarding pain intensity, sleeping, and reading. Conversely, the NDI-5 focuses on function and would require that pain be measured in a different standardized measure, since this is clearly an important issue for people suffering from neck pain. The NDI-5 might allow for clearer distinction between pain and function constructs, but the point at which measures become too short is not clear. Our qualitative work with patients with neck pain suggested that patients want comprehensive consideration of a broad array of life impacts that resulted from neck pain [23].

Finally, there is an update in terms of setting the acceptable level of the local independence which may resulting in the variation of constructing Rasch approved models since the examination of local independence is considered as one important test of assumption under Rasch modelling. Van Der Velde et al. [20] defined the critical residual correlation coefficient should be larger than 0.3 to confirm the presence of LD, where as Walton and MacDermid [24] adopted the criterion of LD being0.2 above the average residual correlation, rather than the straight cuff-off of 0.3 [6, 20, 24]. These methodologic differences may have affected the final versions defined by authors.

Despite the differences in different versions of the NDI and the concerns about the scoring of the full NDI, a benefit of the complete 10 items version is that the score can be transformed into either modified version, whereas this is not the case if either of the 5 or 8 items versions are administered [20, 24].

### Strengths & limitations

The literature review only examined studies published in the English language, which may limit the identification of other potential Rasch solutions of NDI. The study sample was recruited from community clinics in a single city in Canada which restricts the generalizability of study findings.

### Implications

Rasch-based scoring may improve the validity and interpretability of the NDI. Future studies should examine other clinical measurement properties in a head-to-head comparison of the NDI-8 and NDI-5, particularly responsiveness users select between the NDI-5 and NDI-8.

## Conclusion

The traditional NDI-10 should not be used interchangeably with either of two Rasch-approved shorter versions. The conceptual difference between the NDI-5 and NDI should be considered during the decision of NDI-8 and NDI-5.

## Supplementary information

**Additional file 1.** Literature Search within Embase, MEDLINE, PubMed, and Google Scholar

**Additional file 2.** NDI Score Transformation Algorithm

## Data Availability

The datasets during and/or analysed during the current study available from the corresponding author on reasonable request.

## Abbreviations

NDI: The neck disability index
NDI-10: The 10-item version of neck disability index
NDI-8: The 8-item version of neck disability index
NDI-5: The 5-item version of neck disability index
B&A: Bland-Altman
PROM: Patient-reported outcome measure
CTT: Classical test theory
PET: Problem elicitation technique
IRT: Item response theory
DIF: Differential item functioning
PSI: Person separation index
SD: Standard deviation
SE: Standard error
CI: Confidence interval
SDres: SD of the residual
ICF: International Classification of Functioning, Disability and Health

## References

[1] Bland JM, Altman DG (1999). Measuring agreement in method comparison studies. Stat Methods Med Res.

[2] Bland JM, Altman DG (2010). Statistical methods for assessing agreement between two methods of clinical measurement. Int J Nurs Stud.

[3] Bobos P, Macdermid JC, Walton DM, Gross A, Santaguida PL (2018). Patient-reported outcome measures used for neck disorders: an overview of systematic reviews. J Orthop Sports Phys Ther.

[4] Bovim G, Schrader H, Sand T. Neck pain in the general population. Spine. 1994. 10.1097/00007632-199406000-00001. 10.1097/00007632-199406000-00001 8066508

[5] Cano SJ, Barrett LE, Zajicek JP, Hobart JC (2011). Beyond the reach of traditional analyses: using Rasch to evaluate the DASH in people with multiple sclerosis. Mult Scler J.

[6] Christensen KB, Makransky G, Horton M (2017). Critical Values for Yen’s Q 3 : Identification of Local Dependence in the Rasch Model Using Residual Correlations. Appl Psychol Meas.

[7] Covic T, Pallant JF, Conaghan PG, Tennant A (2007). A longitudinal evaluation of the Center for Epidemiologic Studies-Depression scale (CES-D) in a rheumatoid arthritis population using Rasch analysis. Health Qual Life Outcomes.

[8] Croft PR, Lewis M, Papageorgiou AC, Thomas E, Jayson MIV, Macfarlane GJ, Silman AJ (2001). Risk factors for neck pain: a longitudinal study in the general population. Pain.

[9] Evans R, Bronfort G, Schulz C, Maiers M, Bracha Y, Svendsen K (2012). Supervised exercise with and without spinal manipulation performs similarly and better than home exercise for chronic neck pain: a randomized controlled trial. Spine.

[10] Gabel CP, Cuesta-Vargas AI, Osborne JW, Burkett B, Melloh M (2014). Confirmatory factory analysis of the neck disability index in a general problematic neck population indicates a one-factor model. Spine Journal.

[11] Giavarina D (2015). Understanding Bland Altman analysis. Biochemia Medica.

[12] Hogg-Johnson S, Van Der Velde G, Carroll LJ, Holm LW, Cassidy JD, Guzman J (2008). The burden and determinants of neck pain in the general population results of the bone and joint decade 2000-2010 task force on neck pain and its associated disorders task force on neck pain and its associated disorders per-formed a systematic search and. Eur Spine J.

[13] Hoving JL, O’ Leary E, Niere K, Sally G, Buchbinder R (2003). Validity of the neck disability index, Northwick Park neck pain questionnaire, and problem elicitation technique for measuring disability associated with whiplash-associated disorders. Int Assoc Study Pain.

[14] Hung M, Cheng C, Hon SD, Franklin JD, Lawrence BD, Neese A (2015). Challenging the norm: further psychometric investigation of the neck disability index. Spine J.

[15] Iyer S, Koltsov JCB, Steinhaus M, Ross T, Stein D, Yang J (2019). A prospective, psychometric validation of National Institutes of Health patient-reported outcomes measurement information system physical function, pain interference, and upper extremity computer adaptive testing in cervical spine patients: successes and. Spine.

[16] MacDermid JC, Walton DM, Avery S, Blanchard A, Etruw E, McAlpine C, Goldsmith CH (2009). Measurement properties of the neck disability index: a systematic review. J Orthopaedic Sports Physical Ther.

[17] Nazari G, MacDermid JC, Sinden KE, Richardson J, Tang A (2019). Inter-instrument reliability and agreement of Fitbit charge measurements of heart rate and activity at rest, during the modified Canadian aerobic fitness test, and in recovery. Physiother Can.

[18] Packham, T., & Macdermid, J. C. (2013). Measurement properties of the patient-rated wrist and hand evaluation: Rasch analysis of responses from a traumatic hand injury population. J Hand Ther, 26(3), 216–224. dpoi: 10.1016/j.jht.2012.12.006. 10.1016/j.jht.2012.12.006 23561017

[19] Pallant JF, Tennant A (2007). An introduction to the Rasch measurement model: an example using the hospital anxiety and depression scale (HADS). Br J Clin Psychol.

[20] Van Der Velde G, Beaton D, Hogg-Johnston S, Hurwitz E, Tennant A (2009). Rasch analysis provides new insights into the measurement properties of the neck disability index. Arthritis Care Res.

[21] Vernon H (2008). The neck disability index: state-of-the-art, 1991-2008. J Manip Physiol Ther.

[22] Vernon H, Mior S (1991). The neck disability index: a study of reliability and validity. J Manip Physiol Ther.

[23] Vincent JI, MacDermid JC, Ziebart C (2020). Exploratory and Confirmatory factor analysis of the Rheumatoid Arthritis- Work Instability Scale (RA-WIS) in a cohort of workers compensation claimants with upper extremity (In preparation for submission Quality of Life Research) injuries.

[24] Walton DM, MacDermid JC (2013). A brief 5-item version of the neck disability index shows good psychometric properties. Health Qual Life Outcomes.

[25] Wlodyka-Demaille S, Poiraudeau S, Catanzariti JF, Rannou F, Fermanian J, Revel M (2004). The ability to change of three questionnaires for neck pain. Joint Bone Spine.

