# Supplementary material for: Agreement between original and Rasch-approved neck disability index
Source: BMC Med Res Methodol. 2020 Jul 3;20:180. doi: 10.1186/s12874-020-01069-w (PMC7333341; doi:10.1186/s12874-020-01069-w)
Supplement: Supplementary file 1 — Additional file 1. Literature Search within Embase, MEDLINE, PubMed, and Google Scholar [file 12874_2020_1069_MOESM1_ESM.docx]

**Appendix 1. Literature Search within Embase, MEDLINE, PubMed, and Google Scholar**

**Embase** <1974 to 2020 January 24>

**MEDLINE(R)** <1946 to 2020 January 24>

**Search Strategy:**

1 Neck Disability Index/ or NDI.mp.

2 Rasch analysis.mp. or Rasch analysis

3 structural validity.mp.

4 construct validity.mp. or construct validity

5 2 or 3 or 4

6 1 and 5

**PubMed** <1946 to 2020 January 24>

**Search Strategy:**

((Rasch[All Fields] OR (("dna, recombinant"[MeSH Terms] OR ("dna"[All Fields] AND "recombinant"[All Fields]) OR "recombinant dna"[All Fields] OR "construct"[All Fields]) AND validity[All Fields])) OR (structural[All Fields] AND validity[All Fields])) AND ((("neck"[MeSH Terms] OR "neck"[All Fields]) AND Disability[All Fields] AND ("abstracting and indexing"[MeSH Terms] OR ("abstracting"[All Fields] AND "indexing"[All Fields]) OR "abstracting and indexing"[All Fields] OR "index"[All Fields])) OR NDI[All Fields])
