# Supplementary material for: Agreement between original and Rasch-approved neck disability index
Source: BMC Med Res Methodol. 2020 Jul 3;20:180. doi: 10.1186/s12874-020-01069-w (PMC7333341; doi:10.1186/s12874-020-01069-w)
Supplement: Supplementary file 2 — Additional file 2. NDI Score Transformation Algorithm [file 12874_2020_1069_MOESM2_ESM.docx]

**Appendix 2. NDI Score Transformation Matrix**

| NDI 8-item Ordinal | NDI 8-item Linear | NDI 5-item Ordinal | NDI 5-item Linear |
| --- | --- | --- | --- |
| 0 | 0.00 | 0 | 0 |
| 1 | 4.14 | 1 | 8 |
| 2 | 7.15 | 2 | 13 |
| 3 | 9.36 | 3 | 16 |
| 4 | 11.10 | 4 | 19 |
| 5 | 12.53 | 5 | 20 |
| 6 | 13.81 | 6 | 22 |
| 7 | 14.93 | 7 | 23 |
| 8 | 15.89 | 8 | 25 |
| 9 | 16.82 | 9 | 26 |
| 10 | 17.67 | 10 | 27 |
| 11 | 18.45 | 11 | 28 |
| 12 | 19.18 | 12 | 29 |
| 13 | 19.88 | 13 | 30 |
| 14 | 20.57 | 14 | 31 |
| 15 | 21.23 | 15 | 33 |
| 16 | 21.85 | 16 | 34 |
| 17 | 22.47 | 17 | 35 |
| 18 | 23.09 | 18 | 36 |
| 19 | 23.70 | 19 | 37 |
| 20 | 24.28 | 20 | 39 |
| 21 | 24.86 | 21 | 40 |
| 22 | 25.44 | 22 | 43 |
| 23 | 26.02 | 23 | 46 |
| 24 | 26.60 | 24 | 50 |
| 25 | 27.19 |  |  |
| 26 | 27.77 |  |  |
| 27 | 28.35 |  |  |
| 28 | 28.93 |  |  |
| 29 | 29.54 |  |  |
| 30 | 30.16 |  |  |
| 31 | 30.82 |  |  |
| 32 | 31.52 |  |  |
| 33 | 32.17 |  |  |
| 34 | 32.87 |  |  |
| 35 | 33.60 |  |  |
| 36 | 34.42 |  |  |
| 37 | 35.42 |  |  |
| 38 | 36.97 |  |  |
| 39 | 40.72 |  |  |
| 40 | 50.00 |  |  |
| NDI: Neck Disability Index  Ordinal NDI total score is calculated based on original score computation as for each section the total possible score is 5: if the first statement is marked the section score = 0, if the last statement is marked it = 5. Linear NDI total scores are calculated based on the algorithms listed in the publication for NDI 8 and 5-item versions. | | | |
